# Primary Sigmoid Vaginoplasty in Transwomen: Technique and Outcomes

**DOI:** 10.1155/2018/4907208

**Published:** 2018-05-10

**Authors:** Christopher J. Salgado, Ajani Nugent, Joseph Kuhn, Meghan Janette, Heidi Bahna

**Affiliations:** ^1^Division of Plastic Surgery, University of Miami Miller School of Medicine, Miami, FL, USA; ^2^Jacob's School of Medicine and Biomedical Sciences, University at Buffalo, SUNY, Buffalo, NY, USA; ^3^University of Miami Miller School of Medicine, Miami, FL, USA; ^4^Division of Colon and Rectal Surgery, University of Miami Miller School of Medicine, Miami, FL, USA

## Abstract

**Background:**

Many techniques have been described for reconstruction of the vaginal canal for oncologic, traumatic, and congenital indications. An increasing role exists for these procedures within the transgender community. Most often, inverted phallus skin is used to create the neovagina in transwomen. However, not all patients have sufficient tissue to achieve satisfactory depth and those that do must endure cumbersome postoperative dilation routines to prevent contracture. In selected patients, the sigmoid colon can be used to harvest ample tissue while avoiding the limitations of penile inversion techniques.

**Methods:**

Records were retrospectively reviewed for all transwomen undergoing primary sigmoid vaginoplasty with the University of Miami Gender Reassignment service between 2014 and 2017.

**Results:**

Average neovaginal depth was 13.9 +/− 2.0 centimeters in 12 patients. 67% were without complications, and all maintained tissue conducive to sexual activity. No incidences of bowel injury, anastomotic leak, sigmoid necrosis, prolapse, diversion neovaginitis, dyspareunia, or excessive secretions had occurred at last follow-up.

**Conclusions:**

Sigmoid vaginoplasty is a reliable technique for achieving a satisfactory vaginal depth that is sexually functional. Using a collaborative approach, it is now our standard of care to offer this surgery to transwomen with phallus length less than 11.4 centimeters.

## 1. Introduction

Gender affirming surgery is now an established part of the transition experience for transgender patients [1]. These procedures improve quality of life and allow them to participate in relationships that are psychologically and sexually fulfilling [2–5]. Many techniques are used in the creation of the neovaginal canal [1, 6, 7]. Though there is no single optimal technique, inversion vaginoplasty with penile-scrotal flaps is the preferred and most commonly practiced method among surgeons [7]. However, sufficient penile-scrotal skin is not always available because of limitations in either patient anatomy or patient expectations for vaginal depth. Additionally, it is becoming more common for younger patients to undergo hormonal blockade in anticipation of gender transition [8]. Though this forestalls the distressing aspects of going through puberty incongruent with one's gender, it may limit the amount of tissue for penile-scrotal based vaginoplasty. Patients who require revision of a failed primary vaginoplasty encounter a similar problem where sufficient tissue must be derived from elsewhere. Full-thickness skin grafts [9], local flaps, musculocutaneous flaps [10–12], peritoneum [13–15], and various segments of intestinal tissue have been previously described as alternative sources for vaginal reconstruction [16–19].

Intestinal vaginoplasty is a well-described modality for the treatment of congenital or acquired absence of the vagina [20]. In transgender patients, the technique is more often used as a revision procedure after primary failure or complications like vaginal stenosis [21]. Recent analysis of pooled data suggests that patients who undergo intestinal vaginoplasty experience complication and mortality rates comparable with penile inversion vaginoplasty with several advantages [16]. Harvesting the intestinal segment provides for reliable achievement of adequate depth. There are less tendency for intestinal grafts to shrink and therefore less need for lifelong dilation. Additionally, the mucosa feels and appears more like vaginal mucosa with the added benefit of self-lubrication. Performing an elective bowel resection is often perceived as an unnecessary risk to the patient, but recent data suggests that there are fewer gastrointestinal complications in intestinal vaginoplasty than once thought [9, 16]. In this study we present a retrospective series of 12 consecutive patients who underwent primary sigmoid vaginoplasty between 2014 and 2017 at University of Miami Hospital.

## 2. Materials and Methods

A database was created retrospectively to document patients who underwent sigmoid colon vaginoplasty for primary creation of a neovagina between 2014 and 2017 at University at Miami Hospital. Baseline demographics, medical/surgical history, smoking status, complications, and postoperative vaginal depths were collected. Vaginal depth was measured with a dilator and reported in inches. Informed consent was obtained for all patients, including the use of intraoperative photography for publication. This project was granted IRB exempt status.

### 2.1. Preoperative Evaluation

A detailed physical history was taken with special attention to abdominal surgery. In our practice, colonoscopy is recommended for all patients over 40, unless personal or family history indicates otherwise. Elevated BMI was not a contraindication to the procedure. On the morning of surgery or the day before, venous US/Doppler of the upper and lower extremities was performed to rule out deep venous thrombus. Consistent with WPATH guidelines, we recommend that all patients stop estrogen supplementation 2–4 weeks before surgery, and all patients underwent a bowel preparation with GoLYTELY©, Braintree Laboratories, Braintree, MA.

### 2.2. Surgical Procedure

At our institution, laparoscopic sigmoid vaginoplasty is performed in conjunction with a colorectal surgeon, who harvests the pedicled sigmoid conduit for creation of the neovagina. A simultaneous abdominoperineal approach is utilized with the patient in lithotomy position. Perioperative antibiotics are delivered to prevent surgical site infection. An epidural may be placed intraoperatively to assist with postoperative pain.

**Figure 1:**
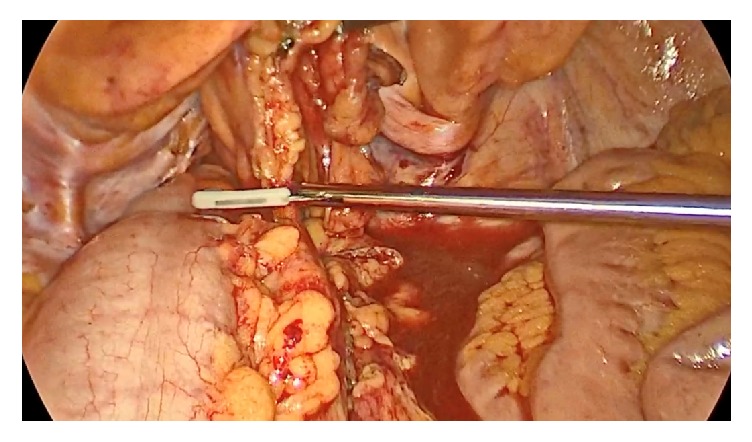
Distracted segment of sigmoid colon with linear staple dissecting it from mesentery at its most lateral extent.

**Figure 2:**
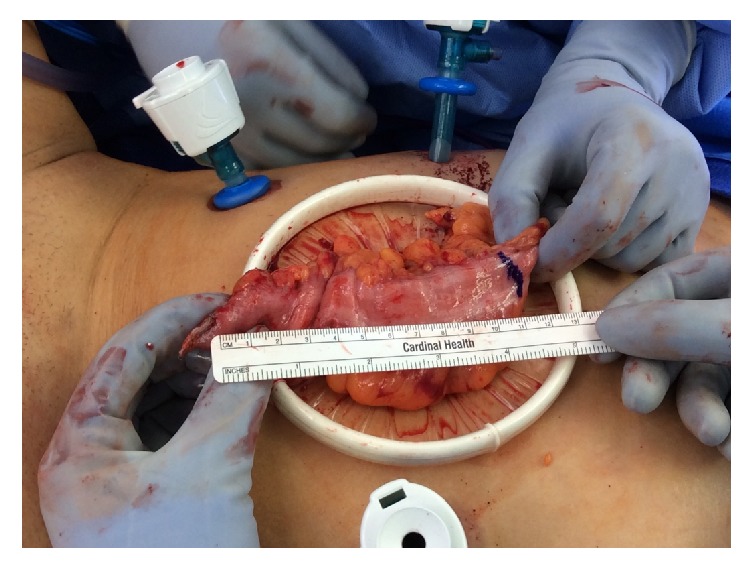
Sigmoid colon segment at the time of laparoscopic harvest.

**Figure 3:**
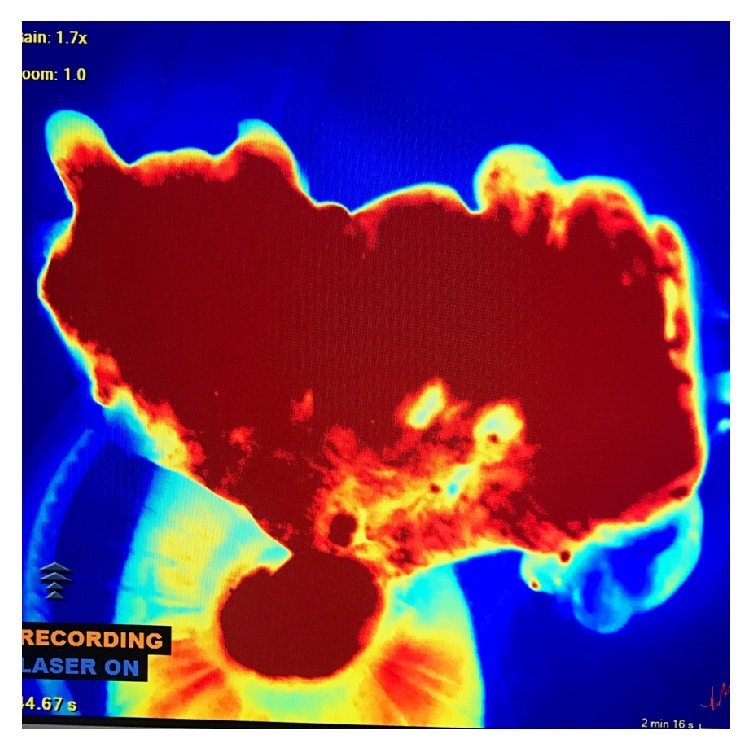
Intraoperative screen capture of extra-abdominal colon segment at the time of laparoscopic harvest using SPY system. Imaging demonstrates abundant perfusion on its pedicle.

The abdominal cavity is accessed through a periumbilical trocar. Pneumoperitoneum is obtained and after no contraindication to proceeding is found, additional trocars are placed. Attention is first directed to the sigmoid colon. Dissection begins lateral to medial along the white line of Toldt. The ureter is identified and retracted. Mobilization of the colon continues up to the splenic flexure using blunt and sharp dissection and the LigaSure device. After adequate mobilization, the colon is medialized. An area of distal sigmoid colon with the longest mesentery is selected to serve as the conduit. A window is created in the adjacent mesentery in order to transect the sigmoid with a linear stapler. The mesentery is further divided along the length of the pedicle while preserving the blood supply to the transected end (Figure 1). The periumbilical incision is extended by 2-3 centimeters. With a wound protector placed, the distal sigmoid is extracorporealized (Figure 2). Proximal to the distal end, a 12–15 cm sigmoidal segment is marked and transected with a linear stapler. Intraoperative injection of indocyanine green and SPY system may be used to confirm perfusion of the sigmoid conduit (Figure 3). The proximal end is prepared for anastomosis by placing the anvil of a circular stapler through the bowel and securing it with a purse string. Visual pulsation of the pedicle to the sigmoid conduit is verified and then returned to the abdominal cavity. The anastomosis is performed with use of an end-to-end circular stapling device. A leak test is performed with the anastomosis submerged in saline and air insufflated into the anus.

**Figure 4:**
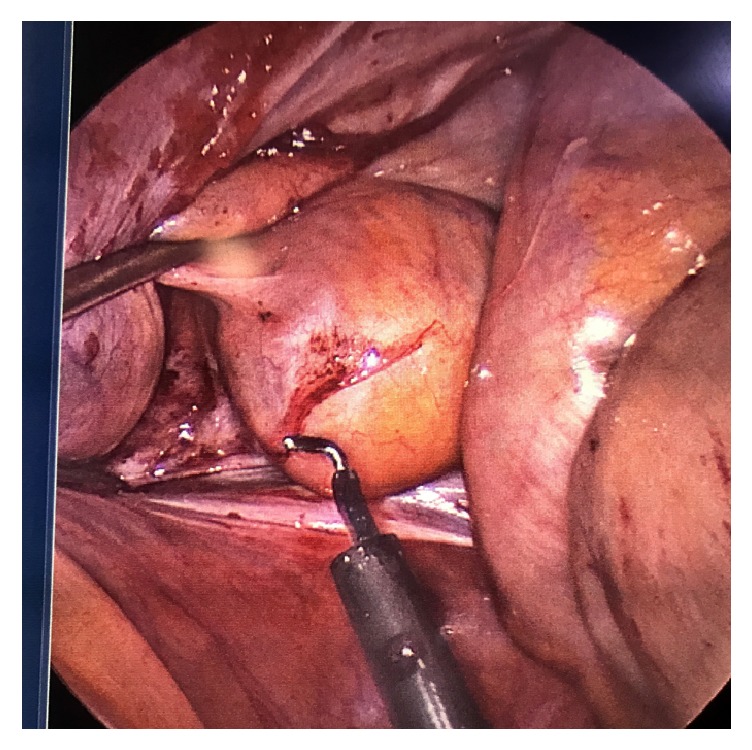
Caudal view of the pelvic cavity showing gentle pressure from the perineal dissection as the peritoneum is opened with electrocautery.

**Figure 5:**
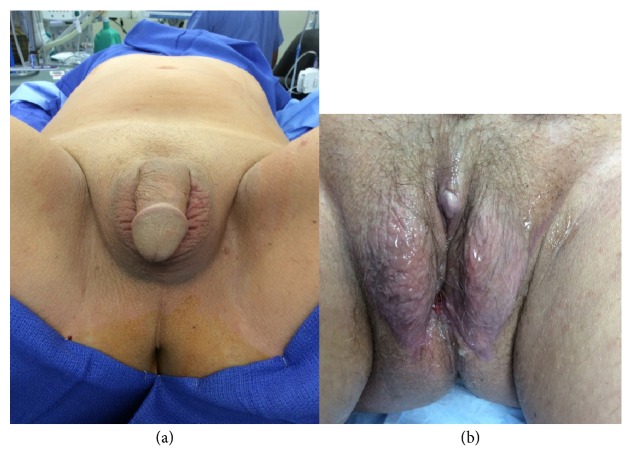
(a) Preoperative image of transwoman in lithotomy position. (b) Postoperative image of transwoman after 6 months. The external genitalia do not differ from traditional penile inversion techniques.

The plastic surgeon begins the primary vaginoplasty and perineal dissection simultaneously. An ellipsoid incision is made with the scrotal raphe midline. Bilateral orchiectomies are performed. At this point they are transected and suture ligated with retraction into the external inguinal ring. The external ring is then closed with absorbable sutures to decrease the risk of an inguinal hernia. The penile skin flap is elevated off the neurovascular bundle and deep underlying corporal tissues. The neoclitoris is harvested from a portion of the glans penis and raised off Buck's fascia under loupe magnification, paying careful attention to harvest all dorsal penile nerves and the deep dorsal artery and veins from the phallus. A Foley is then placed via the corpus spongiosum, which is then dissected from the corpora cavernosa bodies. The corpora cavernosa are further skeletonized proximally to the corporal crura and divided individually with careful suture ligation. The perineal dissection is carried out at the intended posterior fourchette following an inverted U skin design. The dissection is directed to the patient's right to avoid rectal injury. Skin flaps are raised along the inguinal crease for later creation of labia majora tissue. Intra-abdominally, the colorectal surgeon opens the peritoneum with electrocautery while the plastic surgeon unites the abdominal and perineal dissections with gentle traction and electrocautery (Figure 4). The sigmoid conduit is brought through the neovaginal space in an antegrade direction, exteriorized for several centimeters, and inset with minimal tension at the level of the penile stump. Adequate mobilization of the sigmoid is usually achieved by release from lateral attachments and thorough mesenteric dissection. If the segment cannot be transposed tension-free then ligation of the first 1-2 sigmoid arteries and release of accompanying mesentery can further mobilize the sigmoid conduit. The penile skin is then shortened to 1-2 inches to provide for normal appearing external genitalia. Following excision, the penile stump is sutured to the sigmoid conduit with interrupted absorbable sutures. The vascular supply with its mesentery prevents the intestinal segment from prolapsing and allows for a visual appearance like that of a cis-gender vaginal canal. Tissue rearrangement of the scrotal and inguinal skin is performed to contour the labia majora and the urethra is brought just cephalad to the introitus, spatulated, and sutured in place. A clitoroplasty is then performed with a triangular skin incision within the caudal portion of the native mons pubis skin for creation of a clitoral hood. An expander is then placed into the introitus and inflated minimally to avoid compressing the tissues. The final cosmesis of the external genitalia is the same as in penile inversion vaginoplasty (Figures 5(a) and 5(b)).

### 2.3. Postoperative Care and Follow-Up

Patients are admitted to the hospital for 5–7 days, and the condition of the neovagina is checked daily with clear visualization of the intestinal segment. The patient may ambulate after 48 hours of bed rest. If an epidural is used it is discontinued on postoperative days 4–6. The Foley is commonly left in place for ten days and removed in the office. The patient is instructed not to dilate until a follow-up visit and Foley catheter removal.

## 3. Results

12 consecutive patients underwent primary sigmoid colon vaginoplasty from 2014 to 2017. Our patient cohort was on average 47 +/− 15.4 years of age and had a BMI of 26.8 +/− 4.9, and all were white with the exception of one Hispanic patient. Each patient was on a cross-gender estrogen regimen. All patients had an average penis length on stretch of 4.01 +/− 0.76 inches or 10.2 +/− 1.9 centimeters. Overall, 67% (8/12) had no intraoperative or postoperative complications; 6 complications occurred, 4 of which were minor complications (2—ileus, 1—surgical site infection, and 1—intraoperative bladder laceration) and two were considered major complications (1—DVT and 1—suspected PE). There was one return to the operating room (8%) for a suspected intra-abdominal problem, which was negative upon diagnostic laparoscopy and for two patients who underwent secondary revision procedures (17%). Vaginal stenosis occurred in two cases (2 of 12 or 17%) at the neointroitus, which were managed with dilation procedures under anesthesia. A detailed account of complications and their management is available below.

### 3.1. Complications and Hospitalization

A minor bladder injury occurred in one patient. It was repaired intraoperatively through a pfannenstiel incision and the patient recovered without any sequelae. A Foley catheter was left in place for 3 weeks. The average length of stay was 12.5 +/− 9.5 days. This variance was mostly due to one outlier whose long hospital stay was due largely to an anomalous vascular pathology discussed below. Excluding this patient, length of stay was 9 +/− 2.1 days. Two patients developed postoperative ileus that resolved with dietary measures. The patient developed diffuse abdominal pain and leukocytosis on postoperative day 3 and was taken for a diagnostic laparoscopy, sigmoidoscopy, and vaginoscopy that was found to be negative for associated pathology. She received an abdominal washout with continued IV antibiotic treatment and was noted to have symptomatic resolution. One patient developed a deep venous thrombosis of the left external iliac that eventually required thrombolysis, placement of an IVC filter, and stenting for treatment of May-Thurner syndrome, which was discovered during her workup. This prolonged her hospital stay significantly (37 days) but did not compromise the success of her sigmoid vaginoplasty. Her past medical history was significant for a provoked DVT after surgery in the other leg. Her preoperative lower extremity ultrasound was negative for a deep vein thrombosis.

There was one mortality in this series. One patient died from a suspected pulmonary embolism nine days following surgery. A postmortem exam was not requested by the family. This patient had no past history of DVT/PE and discontinued estrogen therapy four weeks before surgery. She received subcutaneous heparin postoperatively for DVT prophylaxis. She could be considered high risk for DVT because she drove >10 hours the day before vaginoplasty and had breast augmentation 24 hours before discharge.

One patient developed a minor surgical site infection 3 weeks after surgery that responded to oral antibiotics. One patient developed mild, while another developed moderate, introital stenosis, 5 and 6 weeks, respectively, after surgery. They were both treated with dilation under anesthesia. Both recovered satisfactory vaginal circumference and continued with dilation regimens. There were no cases of diversion neovaginitis, vaginal prolapse, necrosis of the sigmoid conduit, or rectovaginal fistula in our series.

### 3.2. Outcomes

Average follow-up time was six months by either phone consultation or clinic visit depending on patient distance. The average neovaginal depth at last follow-up was 5.5 +/− 0.8 in. or 13.9 +/− 2.0 cm. 42% of patients reported vaginal intercourse after the procedure, and they all reported pleasurable sensation and satisfaction with their vaginal depth. All achieved vaginal depths conducive to penetrative sex. None of the patients experienced malodorous or excessive neovaginal secretions.

## 4. Discussion

Sigmoid vaginoplasty is a reliable, low morbidity procedure for achieving adequate vaginal depth in the transgender patient [16, 20]. It is our practice to have a careful, informed discussion about our patient's desires for penetrative sex, patient and partner anatomy, and expectations before considering sigmoid vaginoplasty. In our clinic, we tailor the planned vaginal depth to every individual rather than a preconceived ideal. We propose consideration of sigmoid vaginoplasty for patients with less than 4.5 inches or 11.4 centimeters of stretched penile length. This procedure involves releasing a segment of sigmoid colon from its mesentery on the distal sigmoid arteries. Most typically, it is inset in an isoperistaltic fashion and anastomosed with a single line of interrupted sutures to the penile-scrotal elements of the neovaginal canal. Other intestinal conduits have been described, such as the ileum [22–24] and cecum [25], which may preserve the colon's stool reservoir. However, the cecum can be more difficult to inset tension-free given its position and more limited mesentery. Compared with the ileum, the sigmoid colon produces less copious secretions and better approximates vaginal circumference without additional surgical manipulation [6]. The advantages of this procedure over full-thickness skin grafting include reliable creation of vaginal depth, more natural appearing neovaginal mucosa that produces its own secretions, and lower rates of diffuse vaginal stenosis [9]. It is crucial that informed consent explains that the use of colon segments does not eliminate the need for postsurgical dilation. A regimen of dilation is advisable for the first 6–12 months after surgery. However, the goal of dilation is to prevent introital stenosis of the penile-scrotal flaps or penile-colon anastomosis. Long term, patients can usually anticipate less aggressive dilation regimes. Disadvantages include the need for abdominal surgery and bowel anastomosis. Alternatively, omental and peritoneal flaps have been proposed [13–15]. This preserves bowel continuity with the added benefit of reduced operative time and perhaps reduced hospital stays [14, 15]. Omental and peritoneal flaps, however useful, will always require surgical manipulation to tubularize the graft into a neovaginal canal, the healing of which cannot be predicted [15]. Results of peritoneal grafts in transwomen have not been published in peer-reviewed literature. On the other hand, studies have documented the use of the sigmoid for vaginoplasty in transwomen with high rates of sexual and aesthetic satisfaction for the patient [26].

Our retrospective series reports the surgical outcomes of 12 patients undergoing primary sigmoid colon vaginoplasty. The power of our series is limited by its small cohort size (*n* = 12) and by limited follow-up time (6 months). Many of our patients traveled a great distance for the procedure, making long-term clinical follow-up more difficult and burdensome for the patient. Nonetheless, compared to pooled data on this procedure, our technique accomplished reliable, sexually functional neovaginal canals with satisfactory vaginal depth [16]. Postoperative vaginal depth in our series was 5.5 +/− 0.8 inches or 13.9 +/− 2.0 centimeters compared with a range of 11.5–13.0 centimeters [16]. All of our sexually active patients reported sufficient depth for both sexual function and satisfaction. There were two instances of introital stenosis (17%) compared to an 8.6% stenosis rate reported in pooled data [16] and 14.6% in Bouman et al.'s recent series [27]. Both patients were successfully treated with dilation under anesthesia. In our experience, dilation regimens are usually sufficient to relieve this type of stenosis. When stenosis does occur, it normally does so within the first postoperative year [7, 16]. Our limited follow-up time may not have captured every complication or management thereof that may have occurred in this cohort. The rate of complications in our series was 33%, compared with 6.4% in pooled data [16] and 42% [27]. Like Bouman et al.'s recent study, we encountered few intraoperative or postoperative abdominal complications [27]. Clearly, the ability to carry out simultaneous intra-abdominal and perineal operations maximizes visualization and safe retraction of important structures, and this may contribute to lower rates of bowel injury.

Of note, there was one patient mortality in this series from a presumed pulmonary embolism and a deep vein thrombosis in another. The safety and thrombogenesis of hormonal supplementation in transwomen have been the subject of much inquiry [28–30]. WPATH SOC criteria require 12 continuous months of hormone therapy before genital surgery in male-to-female transgender patients [31]. Extensive evidence shows that hormone replacement with estrogen increases the risk for venous thrombosis and pulmonary embolism in cis-gendered women [32]. Some retrospective studies on transwomen demonstrate dramatically increased rates of VTE that approach 20% in those using synthetic estrogens like ethinyl estradiol, a formulation that is no longer recommended [33]. Other studies show no increased risk [29, 30]. Non-first pass route estrogens like transdermal estradiol and estradiol valerate carry lower inherent thrombogenic potential [30, 34]. Epidemiologic research has shown that transwomen may derive estrogen from nonmedical sources, supplement or self-dose prescribed estrogen, use higher risk formulations, and often face barriers to receiving regular follow-up with a health care provider [35]. These factors can lead to supraphysiologic estrogen levels that further increase VTE risk. For these reasons, we recommend discontinuing estrogen therapy 2–4 weeks prior to surgery with resumption only when the patient is ambulatory. Maintaining dialogue with the patient's care team can help monitor estrogen levels. However, there are no tests to monitor synthetic estrogens and no evidence that establishes a risk optimization protocol in transwomen [29, 30, 34]. Both of the aforementioned patients took oral estradiol, stopped estrogen therapy as recommended, and were treated with heparin DVT prophylaxis.

Other known risk factors like obesity were not a factor for these patients, but preoperative venous stasis is a possibility. Given the relative paucity of surgeons well versed in these techniques, many patients must travel long distances pre- and postoperatively. Additionally, there is a short period of bedrest after this procedure that prolongs immobility. The patient mortality in our series underwent breast augmentation on postoperative day 9, which may have further increased her risk. Both patients with thrombotic complications traveled long distances from other states preoperatively. Though there is no data that demonstrates preoperative venous studies are efficacious in reducing DVT or PE risk in transgender patients, we now perform these studies on all patients immediately before surgery. The patient that developed a DVT did so even after instituting this policy. However, given her aberrant venous pathology and past history of DVT, it is difficult to extrapolate her outcome to other patients. Future studies should evaluate estrogen regimens and safety protocols to limit thrombogenic potential in this population.

## 5. Conclusions

Sigmoid vaginoplasty is a reliable technique for achieving satisfactory vaginal depth that is both sexually functional and pleasing to the patient. The procedure is a collaborative undertaking that requires a skilled laparoscopic surgeon, transgender medicine team, and plastic surgeon to work with the patient to optimally achieve their goals. It is now our standard of care to offer this surgery to our transfemale patients with phallus length of less than 4.5 inches or 11.4 centimeters.
